# Pelvic Ring Fracture With Traumatic Testicular Dislocation: A Case Report

**DOI:** 10.7759/cureus.78324

**Published:** 2025-02-01

**Authors:** Shin Nunotani, Kenji Fujita, Hidetoshi Yasutake, Seigo Suganuma, Keito Shimanuki, Satoru Demura

**Affiliations:** 1 Orthopedics, Saiseikai Toyama Hospital, Toyama, JPN; 2 Orthopedic Surgery, Ishikawa Prefectural Central Hospital, Kanazawa, JPN; 3 Orthopedic Surgery, Graduate School of Medical Sciences, Kanazawa University, Kanazawa, JPN

**Keywords:** anterior-posterior compression, emergency urology, male urology, orthopaedics trauma, pelvic ring fracture, trauma and emergency, traumatic testicular dislocation

## Abstract

Pelvic ring fractures are often accompanied by complications, including vascular injuries such as life-threatening intra-abdominal bleeding and urinary tract damage resulting from direct external force. While urethral and bladder injuries are the most common urologic complications, testicular dislocation is rarely reported. Traumatic testicular dislocation (TTD), although uncommon, is frequently associated with motorcycle accidents and remains relatively unfamiliar to orthopedic surgeons, which can lead to delayed diagnosis. However, prolonged untreated dislocation may result in impaired spermatogenesis and endocrine dysfunction. Early detection and prompt urologic consultation are therefore essential. Treatment typically requires careful reduction, and in this case, testicular repositioning was achieved alongside pelvic fracture stabilization. We present a case of pelvic ring fracture complicated by TTD.

## Introduction

Pelvic ring fractures account for 2-8% of all skeletal injuries, with their incidence rising due to an increase in traffic accidents [1,2]. These high-energy traumas are often associated with multiple fractures and organ damage, necessitating a multidisciplinary approach to treatment. Complications can be life-threatening, including intra-abdominal vascular injuries and genitourinary damage that may affect reproductive function [3].

Urogenital injuries occur in over 5% of men and 3.5% of women with pelvic ring fractures [4]. Among these, bladder and urethral injuries are the most common. While the incidence of bladder rupture is similar in both sexes, urethral injuries are significantly more frequent in men [4]. Traumatic testicular dislocation (TTD) is a rare complication of pelvic ring fractures, with limited data available on its incidence.

Here, we report a case involving multiple traumatic injuries, including an anterior-posterior compression type II (APC II) pelvic ring fracture complicated by TTD, which was successfully treated without residual disability.

## Case presentation

**Figure 1:**
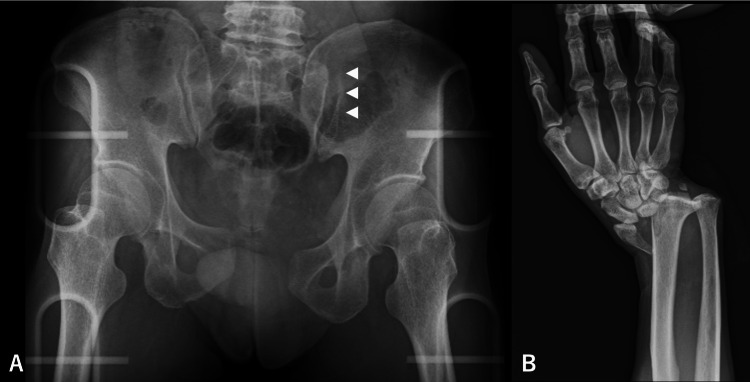
Preoperative X-ray (A) Disarticulation of the left sacroiliac joint (△) and the pubic symphysis. (B) Dislocated fracture of the right wrist joint.

**Figure 2:**
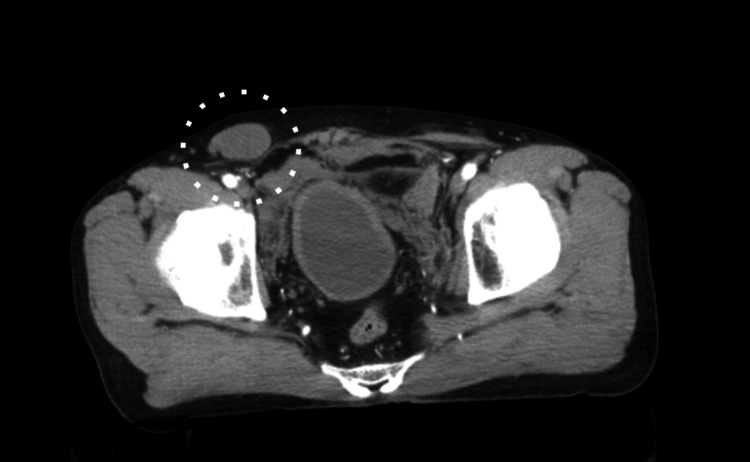
Preoperative axial CT A superficially dislocated right testicle (○) was observed at the level of the acetabulum.

A 55-year-old male presented to the emergency room after colliding with a standard-sized car while riding a motorcycle, wearing a helmet. The patient was in hemorrhagic shock, with a blood pressure of 89/58 mmHg, a heart rate of 107 beats per minute, and an oxygen saturation of 96% on room air, requiring resuscitation with blood products. Imaging examination revealed a dislocated fracture of the right wrist joint, fractures of the right third to seventh ribs with pneumothorax, a fracture of the left fifth lumbar transverse process, an APC II pelvic ring fracture with subluxation of the left sacroiliac joints, and a pubic symphysis avulsion over 2.5 cm. Additionally, the right testis was noted in the right inguinal region, indicative of an undescended testis (Figure 1, Figure 2).

**Figure 3:**
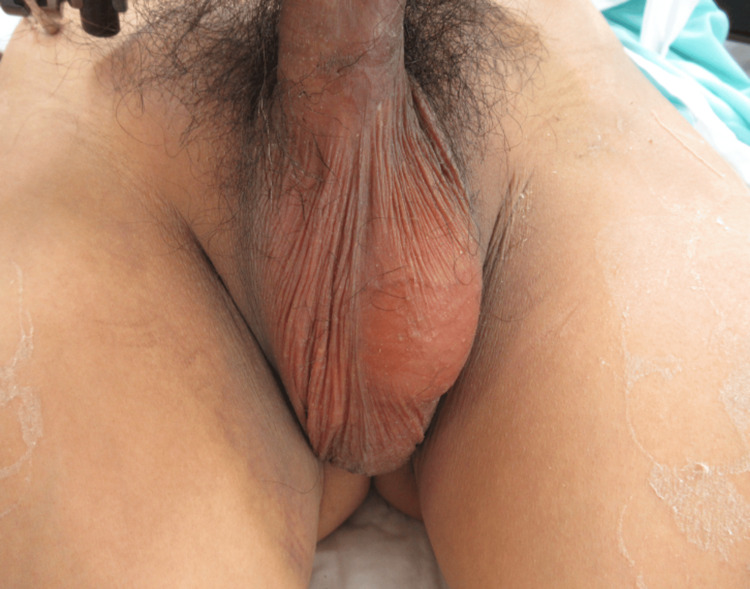
Empty right scrotum with non-palpable testis

On the day of injury, the patient underwent systemic management, including an emergency blood transfusion in the emergency department and external fixation of the wrist joint and pelvic ring using the subcristal method. The right scrotum was empty, and a subcutaneous mass was noted in the right inguinal region (Figure 3). During the subsequent interview, it was revealed that the patient had no history of a retained testicle. Additionally, the patient confirmed that both testicles were in the scrotum before the injury, suggesting that the dislocation may have been triggered by the trauma. The patient was referred to a urologist, who diagnosed TTD.

**Figure 4:**
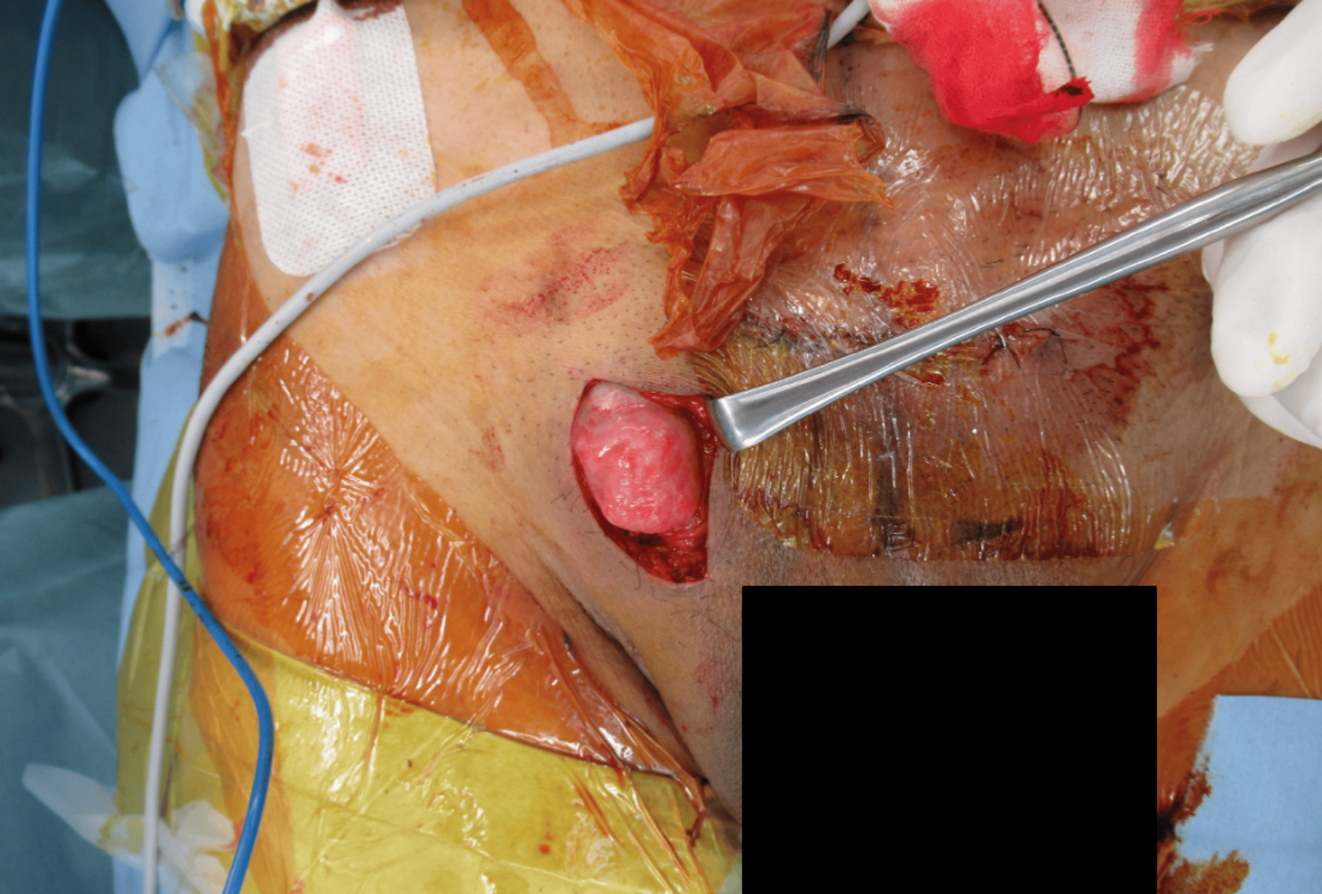
Dislocated testis identified in the right inguinal subcutis

**Figure 5:**
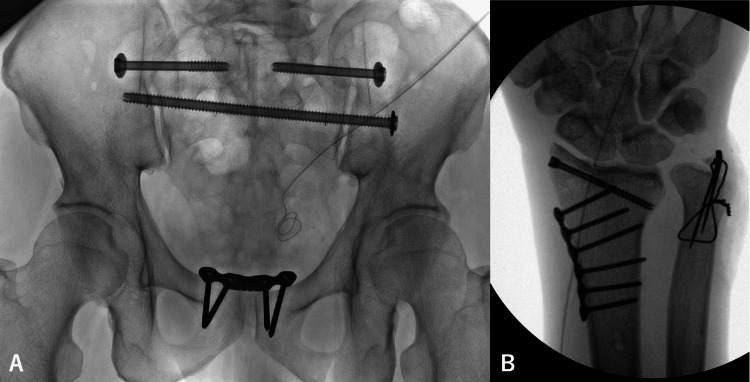
Postoperative X-ray (A) Plate and screw fixation of the pelvis. (B) Plate fixation and pinning of the right wrist joint.

After coordinating with the urologist, the orthopedic surgeon performed radial plate fixation of the wrist and pinning of the ulnar eminence, plate fixation of the pubic symphysis using the Pfannenstiel approach, and iliosacral screw and trans-iliac trans-sacral screw fixation for the posterior pelvis on the 11th day after the accident. During the same operation, the urologist performed an open surgical repair of the testis through a separate incision (Figure 4, Figure 5). As a result, the testis was preserved, and the patient recovered well postoperatively, being able to walk unassisted six months after surgery.

## Discussion

In a report on 180 cases of TTD, Subramaniam et al. found the mean age at the time of injury to be 27 years, with 2.8% of cases involving children under 12 years. Approximately 60% of cases were unilateral, with the most common site of dislocation being subcutaneous in the superficial inguinal region (57.6%), followed by the intra-inguinal canal, intraperitoneal dislocation, pubic region, and perineum, respectively [5].

Straddle injuries, typically occurring when a rider falls onto a convex object and bruises the groin, have long been associated with TTD. More recently, motorcycle accidents have been specifically linked to this type of injury. Testicular dislocation is thought to result from the contraction of the cremaster muscle in combination with direct external force, often caused by the impact of the scrotum with a fuel tank or other object [5].

Pelvic trauma complicating TTD has been reported in association with APC II pelvic ring fractures, as in the present case [6-8], lateral compression-type pelvic ring fractures with T-type acetabular fractures [9], Tile BI pubic fractures [10], and vertical shear-type pelvic ring fractures [11].

TTD is often missed due to the time-sensitive nature of trauma-related fractures and intra-abdominal organ and vascular injuries, which require immediate medical attention [6,12]. In a review of 1,967 male patients with blunt abdominal trauma, nine cases of TTD were identified, all of which were missed on initial examination [13]. Despite this, TTD can typically be diagnosed through simple palpation. If the scrotum is swollen with a hematoma but the testis is not palpable, the possibility of an undescended testis, TTD, or a congenital disorder should be considered, with differentiation made through patient history [5].

CT is also valuable and frequently used by orthopedic surgeons for trauma evaluation. It plays an important role in assessing the scrotum for potential testicular dislocation [5]. Ultrasonography and Doppler ultrasonography can evaluate blood flow and assess the extent of damage, such as testicular rupture [5,6]. In the present case, an undescended testis was initially identified on CT, and TTD was suspected after a subsequent interview revealed no history of undescended testis. This was further confirmed when the patient reported that the testis had been in the scrotum prior to the injury.

If TDD goes undiagnosed and the testis remains dislocated for an extended period, spermatogenesis and endocrine dysfunction may develop. Additionally, there is an increased risk of carcinogenesis, emphasizing the need for early detection and repair of TDD [5,8]. However, delayed detection of hypospermatogenesis has been reported to improve following hematopoietic restoration [14], with some cases recovering after more than 10 years, suggesting that hypospermatogenesis associated with TDD may be reversible [15,16]. In contrast, testicular torsion or rupture requires urgent treatment, highlighting the importance of early detection [7,17].

Treatment for TDD typically involves repairing the scrotum, either manually or surgically. However, manual repair is performed in only 15-39% of cases, with surgical exploration being the preferred approach [18,19]. Scrotal and testicular fixation may be performed to prevent re-dislocation [19]. In the present case, early diagnosis allowed for simultaneous urologic surgery and internal fixation of the pelvic fracture. The patient was successfully treated and showed no signs of residual disability, underscoring the importance of early detection and intervention in such cases.

## Conclusions

TTD is a rare complication of pelvic ring fractures, often overlooked due to its frequent association with other forms of trauma. However, in cases where pelvic ring fractures are caused by trauma exerting strong external force on the perineum, such as motorcycle accidents, it is crucial to thoroughly examine the perineum - preferably through palpation - and conduct imaging tests, including CT and ultrasonography, to rule out TTD. This injury typically requires surgical treatment, which yields the best outcomes when diagnosed early and treated with prompt urologic consultation. With the appropriate clinical management, this condition can be successfully treated without residual disability.
